# Non-pharmacological intervention for shift nurses’ sleep: systematic review and meta-analysis

**DOI:** 10.1093/occmed/kqag085

**Published:** 2026-09-03

**Authors:** A Y L Lee, L A Tse

**Affiliations:** JC School of Public Health and Primary Care, The Chinese University of Hong Kong, Shatin, New Territories, Hong Kong; JC School of Public Health and Primary Care, The Chinese University of Hong Kong, Shatin, New Territories, Hong Kong

## Abstract

**Background:**

Nurses working rotating shifts often experience poor sleep quality due to circadian rhythm disruption. Previous reviews primarily focused on managerial changes to improve nurses’ alertness during night shifts. Although some studies found that individual-level non-pharmacological interventions may significantly alleviate sleep problems, past reviews focused only on aromatherapy.

**Aims:**

To understand current trends and synthesise the effectiveness of non-pharmacological interventions for improving shift nurses’ sleep quality.

**Methods:**

A systematic review and meta-analysis were performed using seven databases for experimental trials published from 2014 to 2024 examining the effectiveness of non-pharmacological interventions on improving shift nurses’ sleep quality. The methodological quality of the included studies was assessed by Cochrane Risk of Bias 2 (RoB 2) tool. Meta-analysis was performed under fixed or random effect model according to heterogeneity assessed by *I*^2^ statistics. Sensitivity analysis and subgroup analysis were also conducted.

**Results:**

A total of 15 articles were included in the narrative synthesis, and 11 of them were included in the meta-analysis. Mindfulness programs were the most common intervention, followed by aromatherapy. Overall pooled analyses of 11 studies showed a significant improvement in shift nurses’ sleep quality with a standardised mean difference (SMD) of −0.63 (95% CI −1.01 to −0.25; *P *= 0.001; *X*^2^ = 71.53; *I*^2^ = 86%; *P *< 0.00001). Mindfulness programs were found to be beneficial, but no significant improvement was noted for aromatherapy.

**Conclusions:**

Non-pharmacological interventions could effectively improve shift nurses’ sleep quality. Mindfulness programs are gaining popularity and were shown to be beneficial but need to be further improved with personalised counselling to improve compliance with irregular work schedules.

Key learning points **What is already known about this subject:**

- Previous on increasing nurses’ alertness during night shifts and minimising fatigue-related medical incidents through institutional approaches, such as work schedule amendment and nap breaks.

**What this study adds:**

- Mindfulness among shift nurses after COVID-19 pandemic, circumventing the barrier of social distancing.
- Non-pharmacological interventions, especially mindfulness programs, were found to be effective to improve shift nurses’ sleep quality.
- Aromatherapy could not significantly improve shift nurses’ sleep quality in this meta-analysis, which is inconsistent with a previous review, possibly due to the use of a different aroma regime.

**What impact this may have on practice or policy:**

- Policymakers in different countries could consider promoting non-pharmacological interventions (e.g. mindfulness programmes) to alleviate shift nurses’ sleep problems and hence improve medical service quality.
- Future research is needed to design a new assessment tool for sleep quality specifically for shift workers to accommodate their shift schedule during assessment.

## Introduction

Nurses were one of the largest working populations engaged in rotating shifts and suffering from poor sleep quality, especially during the COVID-19 pandemic [1]. According to a meta-analysis, around 61% of shift nurses were experiencing shiftwork-related sleep problems globally [2]. Irregular sleep patterns, altered physical activities and food intake pattern, could disturb shift nurses’ circadian rhythm, which regulates sleep and wake cycles, especially during night shifts, and leading to poor sleep quality [3]. Sleep quality was defined as one’s sleep experience assessed both subjectively and objectively [4]. Poor sleep quality could lower shift workers’ health status [5, 6], immunity [7], mental health [8], quality of life [9], and increase the risk of developing different chronic diseases [10, 11], such as cardiovascular diseases [12], diabetes [13], hypertension [14], and increased all-cause mortality [15]. Sleep deprivation was also shown to impair shift nurses’ attention, memory and job performance [16], contributing to medical incidents and long-term job burnout [17]. Therefore, sleep quality and sleeping disorders among shift nurses were growing concern and require immediate public health actions.

Current evidence suggested both pharmacological and non-pharmacological interventions to cope with poor sleep quality among shift nurses. Medications, such as melatonin, a hormone regulating sleep-wake cycle, were found to improve shift nurses’ sleep quality [18]. Non-pharmacologically, most prior research focused on managerial interventions, such as amending shift schedule, providing nap breaks during night shifts, and adjusting work environment with bright-light exposure, aiming to increase nurses’ alertness during night shift and minimising fatigue-related medical incidents [19]. In fact, apart from institutional approaches, individual-level interventions were also important to improve sleep quality. For example, aromatherapy was becoming more popular among shift nurses and proven to be effective on improving their sleep and relieving stress [20, 21], by stimulating the secretion of melatonin or other sleep-related neurotransmitters [22]. It was convenient to be applied through inhalation, massage or simply putting it near their bed [23]. Other non-pharmacological interventions, such as sleep hygiene training, cognitive behavioural therapy for insomnia (CBT-I), and physical exercises, were also developed recently to alleviate sleep problems among shift nurses [24, 25].

To the best of our knowledge, only one systematic review, published in year 2020, was available to understand the effectiveness of non-pharmacological interventions for improving shift nurses’ sleep quality [25]. It included 13 experimental studies but focused on the significant improvement in shift nurses’ sleep quality after receiving aromatherapy with a pooled standardised mean difference of 0.97 (95% CI 0.64–1.29). Also, only studies published before 2018 were included and other non-pharmacological interventions (e.g. CBT-I, education program, exercise training) were not evaluated. New online sleep hygiene programs developed during the COVID-19 pandemic were also not included. Therefore, a new review was needed to evaluate the most updated and more types of non-pharmacological interventions.

Therefore, this review aimed to address the research gap in previous research by capturing more updated experimental trials evaluating the effectiveness of non-pharmacological interventions on improving shift nurses’ sleep quality, including those developed during the COVID-19 pandemic. If these interventions were proven to be effective, the findings could help policymakers to identify ways to alleviate sleep problems at the individual level, instead of managerial changes only. This review also aimed to evaluate the limitations of current non-pharmacological interventions to provide insights for future improvement and development.

The objectives of this review were: (1) to review the current trends in non-pharmacological interventions for improving sleep quality among shift nurses and (2) to synthesise the effectiveness of current non-pharmacological interventions for improving shift nurses’ sleep quality.

## Methods

A systematic review and meta-analysis were conducted following the Preferred Reporting Items for Systematic Reviews and Meta-Analyses (PRISMA) 2020 statement. This study was registered in the International Prospective Register of Systematic Reviews—PROSPERO (CRD420251042304).

A total of seven electronic databases, including PubMed, MEDLINE (via Ovid SP), CINAHL Ultimate (via Ebscohost), EMBASE (via Ovid SP), PsycINFO (via Ovid SP), Scopus and Cochrane Central Register of Controlled Trials (CENTRAL), were the information sources of this review. The searching time frame was 10 years from year 2014 to 2024 to capture the most updated literature. Handsearching was conducted through Google Scholar and reference lists of included papers. The searching strategies were constructed based on three key search terms, including ‘sleep quality,’ ‘nurses’ and ‘shift work,’ and were further expanded by MeSH terms corresponding to each database. More detailed searching strategies are shown in Supplementary File S1.

Articles had been screened using the below eligibility criteria framed by PICOs strategies. The definition of shift work from the National Sleep Foundation was adopted, in which shift work referred to any working schedule that includes working hours outside 7 a.m. to 6 p.m [26]. Rotating shifts were defined as shift work patterns that required nurses to cycle through different working hours, such as morning, evening, day or night shifts and may change every day, week or month. For inclusion criteria, articles were included if all of the following criteria were fulfilled: (1) Participants (P): nurses working on rotating shifts with no limitation of their working institutions (e.g. hospitals, nursing homes), (2) Interventions (I): one of the intervention arms should be non-pharmacological intervention aiming to improve sleep quality among shift nurses, (3) Comparisons (C): control groups with no intervention, placebo control or waitlist control (participants who would receive the intervention after the research ended), (4) Outcomes (O): sleep quality with no limits of subjective or objective measurement and (5) Studies (S): studies adopted experimental trial designs, available in full text, and written in English. For exclusion criteria, articles were excluded if any of the following criteria were met: (1) Participants (P): studies including other shift workers as participants and (2) Interventions (I): interventions including the use of medication for improving sleep quality and interventions focusing only on reducing sleepiness during night shift or on managerial level (e.g. shift schedule changes).The whole literature screening procedure and reasons for exclusion were reported using PRISMA 2020 flowchart. Search results from the databases were retrieved and imported into EndNote 20, removing duplicates. Articles were screened by title and abstract, followed by full text, while articles from handsearching were screened by full text directly.

After literature screening, the following variables in all eligible studies were extracted to a pilot-tested data extraction table: study characteristics (author, year of publication, study design, study location, study population, study duration), intervention characteristics (intervention group, control group), outcome (data collection timepoint, outcome measures, results), limitations, and recommendations mentioned.

The Cochrane Risk of Bias 2 (RoB 2) tool was used to assess the methodological quality of all included studies [27]. It consisted of five domains: (1) risk of bias arising from the randomisation process, (2) risk of bias due to deviations from the intended interventions, (3) missing outcome data, (4) risk of bias in measurement of the outcome and (5) risk of bias in selection of the reported result. The overall risk of bias for each study was categorised as low risk (low risk of bias for all domains), high risk (high risk of bias for either one domain or some concerns for multiple domains) or some concerns (some concerns for either one domain) based on the grading of the above five domains. Two reviewers performed quality appraisal independently and resolved any disagreements through discussion.

Narrative synthesis was performed guided by the Cochrane Handbook [27], summarising and exploring the current trends of various types of non-pharmacological interventions based on the tabulated data extracted. A meta-analysis was conducted in RevMan Web to determine pooled effect measures for overall effectiveness and each type of non-pharmacological intervention at the longest follow-up timepoint, with results illustrated by forest plot if at least two studies examined the same type of intervention. Heterogeneity of the studies included was assessed by chi-squared test, *I*^2^ statistics and *P* value (< 0.05). The *I*^2^ statistics was interpreted as unimportant (0 − 40%), moderate (30 − 60%), substantial (50 − 90%) or considerable (75 − 100%) heterogeneity [27]. Random effect model was used in the meta-analysis if *I*^2 ^> 50%, otherwise fixed effect model was applied. Mean difference (MD) (if same measurement tool) or standardised mean difference (SMD) (if different measurement tools) and 95% confidence interval were computed for continuous variables, while odds ratios (OR) and 95% confidence interval were calculated by Mantel − Haenszel method (fixed effect model) or DerSimonian and Laird method (random effect model) for dichotomous variables. By following Cohen’s rule of thumb, the effectiveness of intervention was interpreted according to its SMD with small (0.2 − 0.5), moderate (0.5 − 0.8) or large (> 0.8) effect. Sensitivity analysis was conducted by repeating the analysis again excluding studies with high risk of bias as assessed by the Cochrane RoB 2 tool. Also, publication bias was evaluated by visualisation of funnel plot, however, Egger’s test was not performed due to high heterogeneity discovered in overall pooled meta-analysis. Ethical approval was not required.

## Results

The screening process was reported using PRISMA 2020 flowchart (Figure 1). A total of 49364 articles from database searching and four articles from handsearching were identified after removing duplicates for title and abstract screening. Of these, 91 articles were screened by full-text and 14 of them were included. Due to the large number of articles available for screening, a second-round search in the seven databases and handsearching was performed for each intervention type to ensure no eligible articles were missed and to reduce bias. At last, 15 articles met the eligibility criteria for narrative synthesis and 11 of them had data available for meta-analysis. The characteristics of all included studies were presented in a data extraction table (Supplementary File S2).

**Figure 1:**
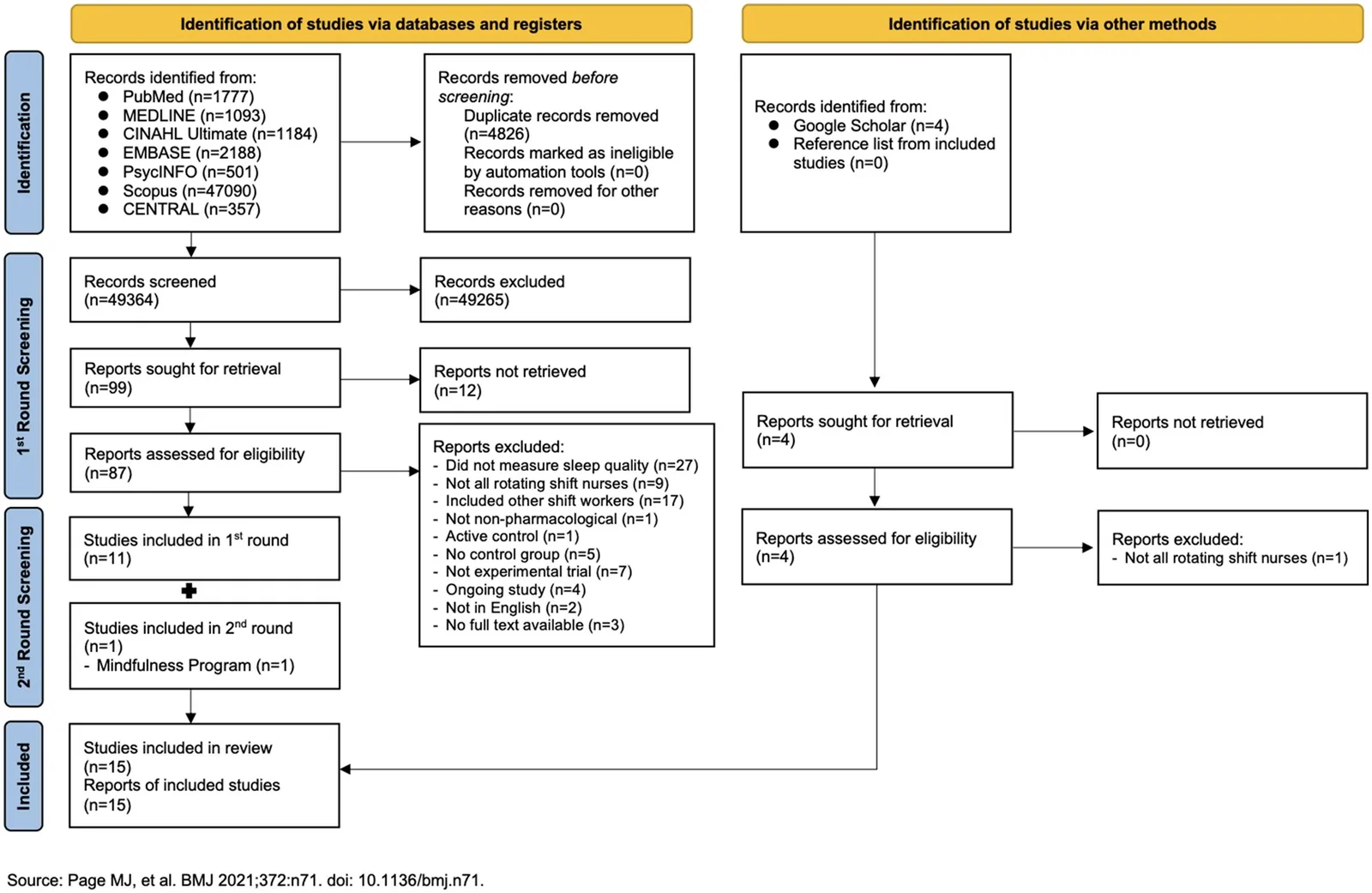
PRISMA 2020 flowchart.

There were 13 randomised controlled trials [28–40] and two quasi-experimental trials [41, 42] included in this review. These studies were conducted in different countries, including Iran [34, 37–39], Korean [35, 41], Jordan [28, 36], Australia [29], Taiwan [30], Canada [31], Sweden [32], Germany [33], China [40] and Indonesia [42]. A total of 1235 shift nurses with mean age ranging from 27 to 35 years old were included in these 15 studies, in which 622 and 613 of them were allocated in intervention and control group, respectively. All shift nurses worked in hospitals and five studies only included those suffering from insomnia [30, 31, 33, 39, 40].

A brief description of intervention characteristics of the included studies was summarised in Table 1 (available as Supplementary data at *Occupational Medicine* Online). Overall, mindfulness programs [28, 32, 33, 36, 38, 40, 42] (*n* = 7, 46%) were the most commonly adopted non-pharmacological interventions, followed by aromatherapy [30, 34, 37] (*n* = 3, 20%), exercise training [35, 41] (*n* = 2, 13%), sleep hygiene education program [29, 39] (*n* = 2, 13%) and light therapy [31] (*n* = 1, 6%). Five studies were implemented through online platforms [33, 35, 38, 40, 41], using Internet-based materials, smartphone and videoconferencing. The included studies were published between year 2016 to 2024, with mindfulness program being more popular in recent years, while aromatherapy being more prevalent before 2017 (Supplementary File S3). No studies were found between 2018 and 2020 due to COVID-19 pandemic. The study duration ranged from 20 days to 18 weeks, with 4 weeks (*n* = 5) being the most common.

The most common intervention was mindfulness programs with a total of seven studies included, in which three of them were delivered online [33, 38, 40]. These programs integrated education (*n* = 9), mind-body therapies (e.g. meditation, body scan, stretching exercises) (*n* = 6) [28, 33, 36, 38, 40, 42], cognitive behavioural therapy for insomnia (*n* = 3) [32, 33, 40], and motivational interviewing (*n* = 1) [32], aiming to improve shift nurses’ sleep quality by relieving stress and encouraging healthy sleep hygiene practices. Some studies supplemented written materials or videos after sessions to facilitate home practices [28, 32, 38, 40], and provided follow-up support through text-messages or automatic reminders [28, 32, 33, 38, 40].

Three studies tried to alleviate shift nurses’ sleep problems with aromatherapy using different types of aroma essential oil made from sweet marjoram [30], lavender essential oil [34], and *P. graveolens* essential oil [37]. Two studies applied aromatherapy through inhalation, instructing shift nurses to carry the prepared aroma essential oil in a container [34] or drop aroma oil on their mask [37] for inhaling its scent when working, while one study combined aroma inhalation and massage in their intervention [30].

Two studies delivered exercise training programs through online platforms using mobile apps [41] and videoconferencing [35]. Both studies encouraged exercise training at home supported with instructional videos uploaded online, while one study conducted live group exercise sessions with real-time feedback from instructors [35]. Text-messaging follow-up was also provided in both studies to monitor shift nurses’ progress and encourage exercise habits.

Two studies conducted education programs aiming to improve shift nurses’ sleep quality by enhancing their knowledge on healthy sleep hygiene practice (e.g. napping, avoiding caffeine consumption). One study provided an 8-weeks training program [29], while another study delivered only one single session [39].

Only one study implemented light therapy to improve shift nurses’ sleep quality by adjusting their circadian rhythm, however, no significant improvement was found [31].

Sleep quality was primarily assessed using subject measures, with Pittsburgh Sleep Quality Index (PSQI) (*n* = 9) and Insomnia Severity Index (ISI) (*n* = 6) being the most frequently used scales in the included studies. In contrast, only one study included objective measures, actigraphy, for evaluating shift nurses’ sleep quality [33]. The PSQI was a 19-items questionnaire including seven components, with total score ranged from 0 to 21. It aimed to assess one’s sleep quality in the past month, with a score higher than 5 indicating poor sleep quality [43]. The ISI was a 7-items questionnaire rated with 5-points Likert scale, measuring one’s subjective perception and consequences regarding their insomnia symptoms over one month [44].

Pooled intervention effect of 11 studies reflected a significant improvement of sleep quality in intervention groups (SMD = −0.63; 95% CI −1.01 to −0.25; *P *= 0.001) after receiving non-pharmacological interventions, but with significant heterogeneity (*X*^2^ = 71.53; *I*^2^ = 86%; *P *< 0.00001) under random effect model (Figure 2). Sensitivity analysis was conducted by removing three studies with high risk of bias. Both result and heterogeneity did not change significantly (SMD = −0.48; 95% CI −0.85 to −0.11; *X*^2^ = 37.28; *I*^2^ = 81%; *P *< 0.00001). Subgroup analysis was also performed due to high heterogeneity (Supplementary File S4). No significant subgroup effect was seen when examining shift nurses having or not having sleep problems (*X*^2^ = 0.41; *I*^2^ = 0%; *P *= 0.52) within different years of publication (*X*^2^ = 0.06; *I*^2^ = 0%; *P *= 0.80). Whereas a statistically significant subgroup effect was found between different outcome measurement tools (*X*^2^ = 9.00; *I*^2^ = 77.8%; *P *= 0.01).

**Figure 2:**
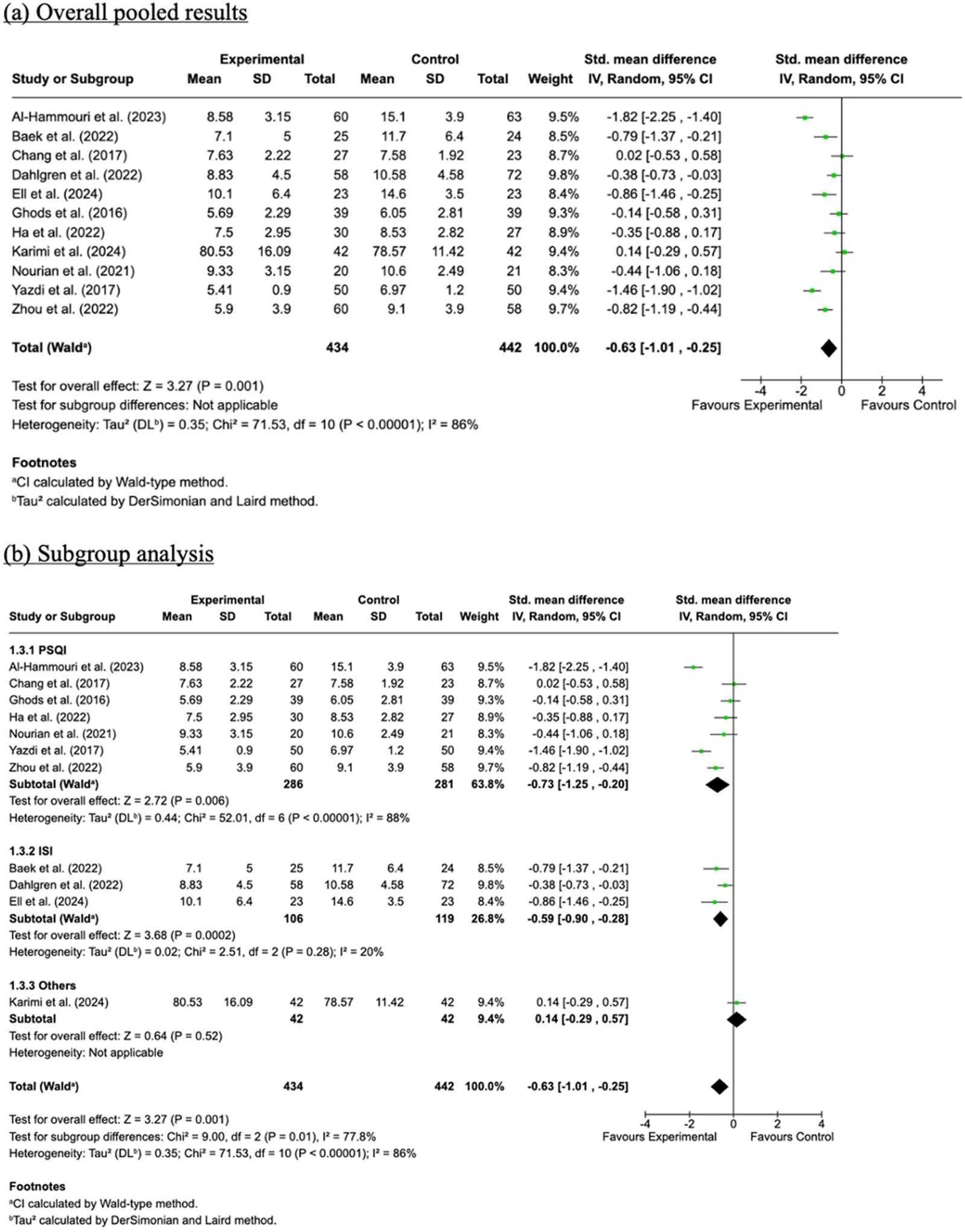
Forest plot for (a) overall effectiveness of non-pharmacological interventions, (b) subgroup analysis according to outcome measurement tools.

Meta-analysis was also performed for different types of non-pharmacological interventions used (Figure 3). Among studies of adopted mindfulness programs, six of them reported significantly better sleep quality among shift nurses in intervention groups when compared to those in control groups. The remaining study showed no significant difference between groups. Although only five of these studies could be included in meta-analysis due to data incompleteness, mindfulness programs were shown to be effective on improving shift nurses’ sleep quality (SMD = −0.90; 95% CI −1.41 to −0.38; *P *= 0.0007; *X*^2^ = 25.83; *I*^2^ = 85%; *P *< 0.0001) in random effect models. For aromatherapy, a meta-analysis of three studies showed no significant difference in sleep quality between shift nurses receiving aromatherapy and placebo (SMD = 0.01; 95% CI −0.26 to 0.28; *P *= 0.94; *X*^2^ = 0.78; *I*^2^ = 0%; *P *= 0.68) in fixed effect models. Although only two studies were available for exercise training, a meta-analysis was performed and revealed a positive effect on improving sleep quality among shift nurses (SMD = −0.55; 95% CI −0.94 to −0.16; *P *= 0.006; *X*^2^ = 1.20; *I*^2^ = 17%; *P *= 0.27) in fixed effect models. Comparing the above, the effect size of mindfulness program (SMD = −0.90; 95% CI −1.41 to −0.38) was relatively higher than aromatherapy (SMD = 0.01; 95% CI −0.26 to 0.28) and exercise training (SMD = −0.55; 95% CI −0.94 to −0.16). While their 95% CI were highly duplicated, the effect size difference between different types of intervention were not statistically significant.

**Figure 3:**
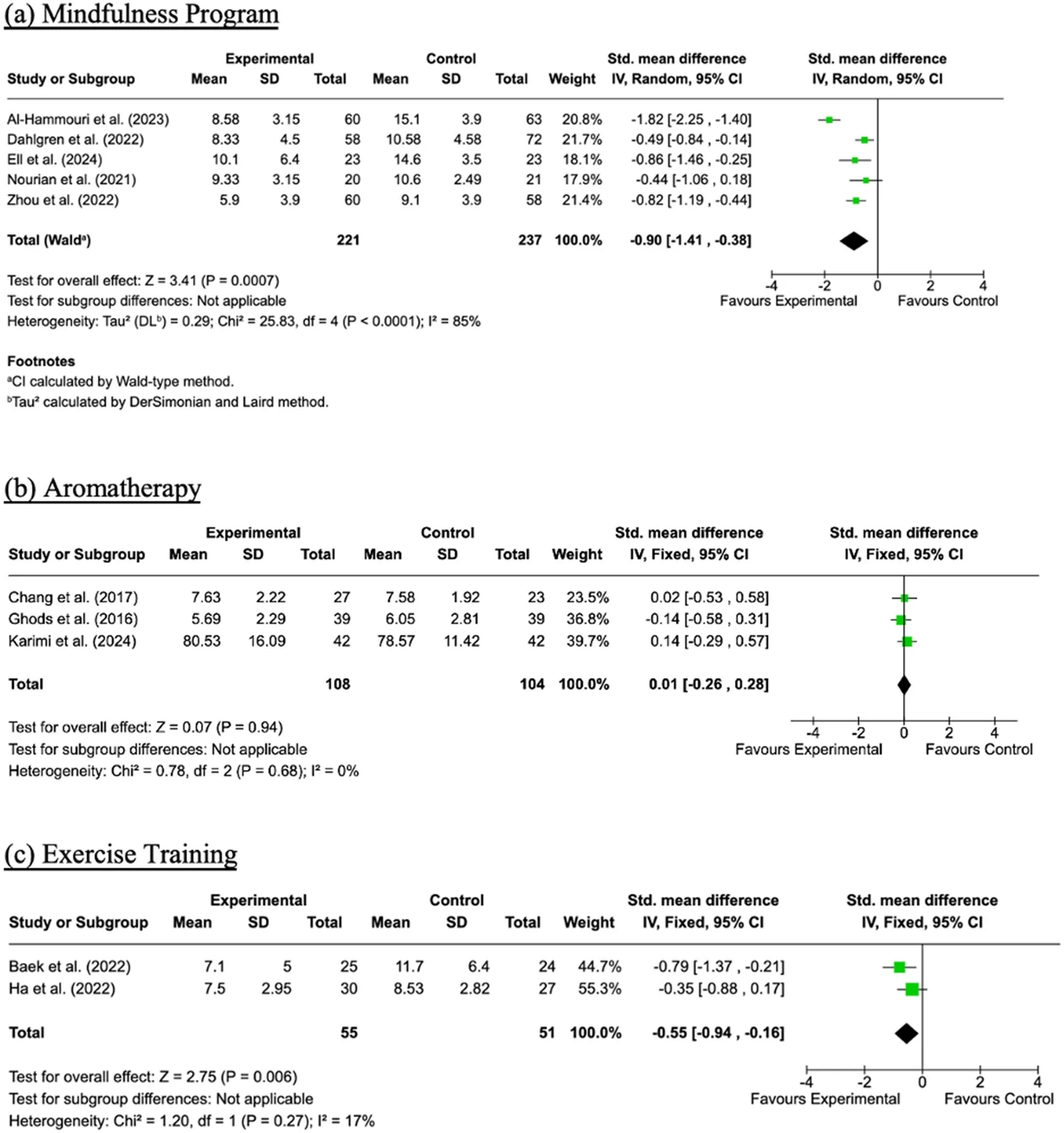
Forest plot for (a) mindfulness program, (b) aromatherapy and (c) exercise training.

The risk of bias of included studies was summarised in Figure 4. Overall, only two studies were rated with low risk of bias (13%), nine studies were rated with some concerns of bias (60%), and the rest were graded with high risk of bias (26%). Most studies had low risk of bias from missing outcome data (D3) and selection of the reported result (D5). However, except for the two quasi-experimental studies, some concerns were seen on the randomisation process as some studies did not mention details regarding allocation concealment. In addition, due to the nature of intervention, blinding of participants, interventionists and assessors may be difficult. Moreover, a test of publication bias was performed by generating a funnel plot for all studies included in meta-analysis (Figure 5). This was relatively symmetrical, indicating the absence of publication bias.

**Figure 4:**
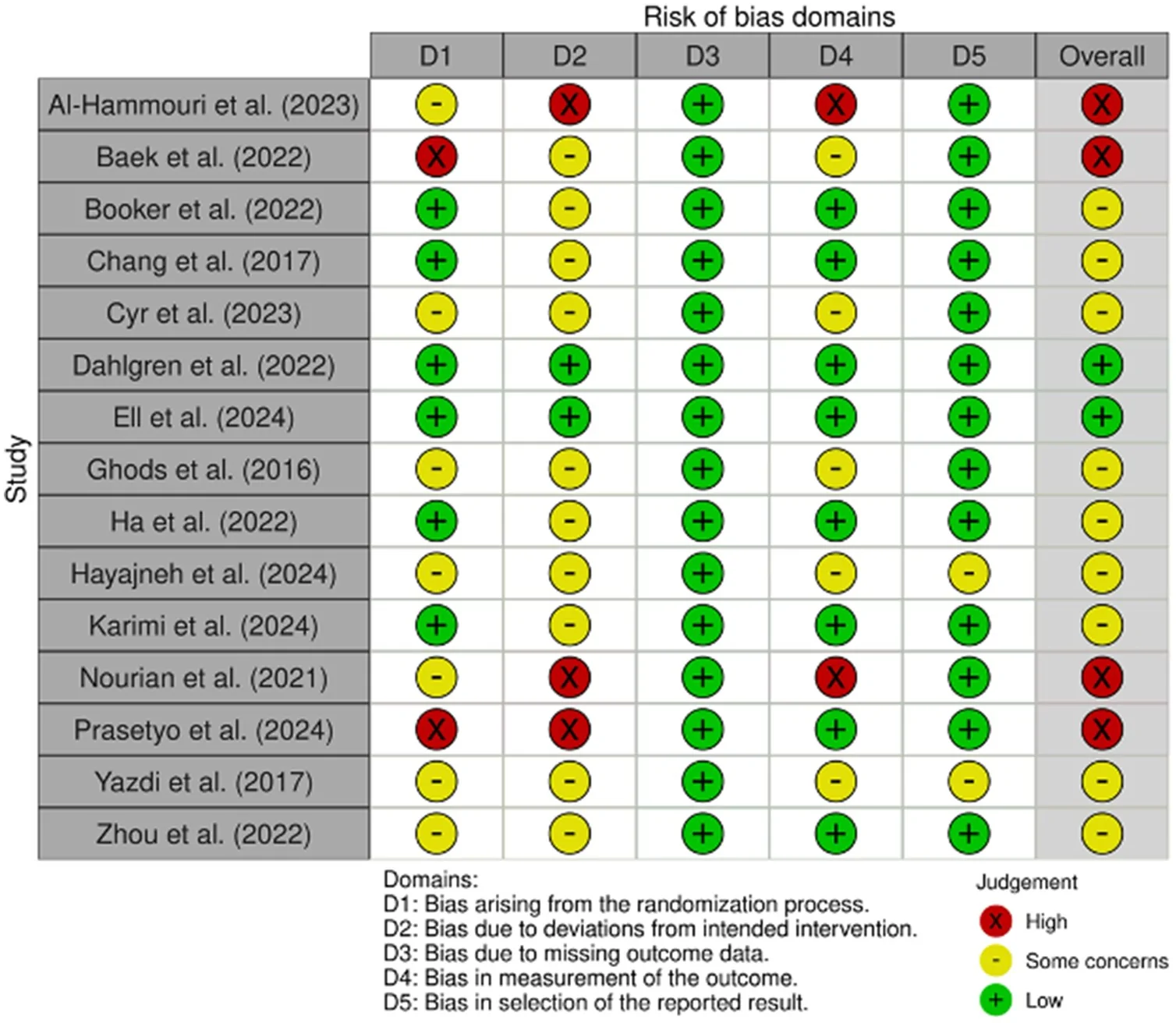
Risk of bias of included studies.

**Figure 5:**
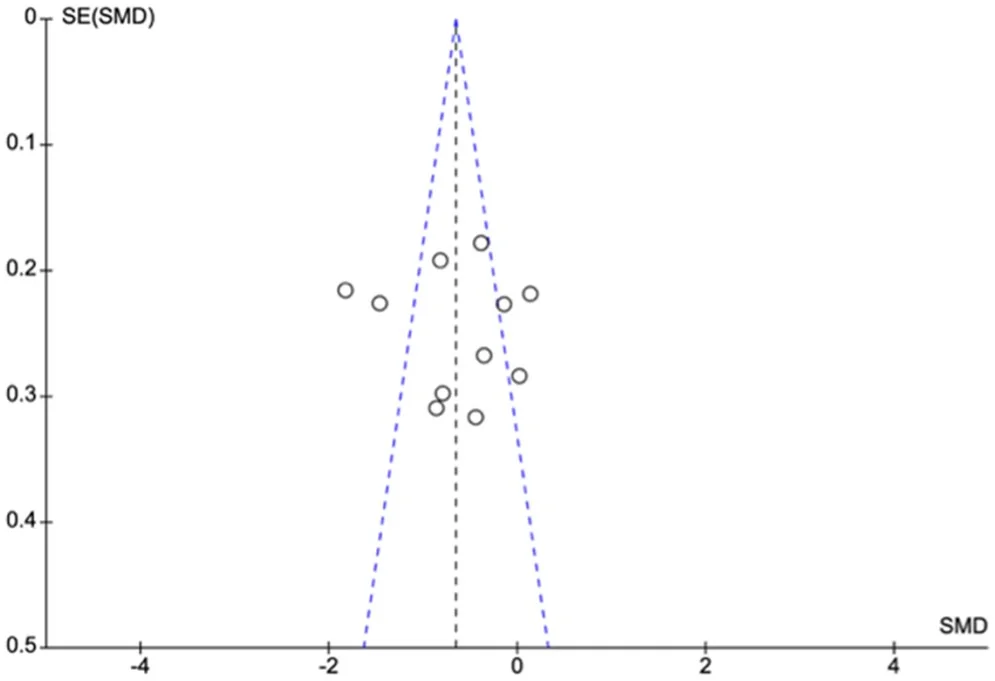
Funnel plot of standardised mean difference against standard error.

## Discussion

This study reviewed 15 experimental trials published from 2016 to 2024 and showed that mindfulness programs were the most adopted non-pharmacological intervention to improve shift nurse’s sleep quality. Moreover, this meta-analysis, including 11 studies, found that various non-pharmacological interventions were an effective way to improve shift nurses’ sleep quality under irregular shift schedule and work stress. Overall, non-pharmacological interventions, especially mindfulness programs, were found to be effective in improving their sleep quality. Although exercise programs also reflected a positive effect, only two studies were available to be included. Moreover, inconsistent with previous review [25], no significant improvement on sleep quality was noted among shift nurses after receiving aromatherapy in this meta-analysis.

Current mindfulness programs had improved shift nurses’ sleep quality by integrating mind-body therapies or cognitive behavioural therapies [45], which had fewer adverse effects than medications [46]. Mind-body interventions, such as meditation, breathing exercises and stretching, could help to improve one’s sleep quality by relieving physical tension and reducing intrusive thoughts [47], while cognitive behavioural therapies focused on facilitating shift nurses’ behavioral changes related to sleep hygiene [46]. A previous meta-analysis also found that mindfulness programs significantly improved sleep quality across different shift workers [48]. Despite these positive results, some studies reported that the benefits of mindfulness programs were hindered by low compliance [32] and high drop-out rates [49], as most of the existing programs were implemented through regularly scheduled sessions and follow-up, which could be difficult to align with the diverse rotating shift schedule for all nurses [33, 50]. Although some studies provided online materials for self-practising mindfulness exercise at home after each session, its positive effect could be reduced as participants could not attend all sessions and eventually lost their enthusiasm. Therefore, future research should focus more on evaluating the effectiveness of online, self-paced mindfulness programs delivered through smartphone apps, allowing shift nurses to accommodate sleep hygiene practice according to their shift schedules. Thereby increasing their willingness to comply with mindfulness exercises and reducing drop-out rates [28, 38, 51]. Follow-up support for practice reinforcement was also needed to monitor participants’ progress throughout the program so that its long-term benefits could be sustained [49]. Moreover, most of the existing mindfulness programs only focused on either cognitive behavioural therapy or mediation exercises, which could not be comprehensive enough to maintain long-term improvement of sleep quality. By incorporating education, behavioural therapy and exercise training components, the content of mindfulness program could be more extensive and have better effects on promoting both lifestyle modification and sleep hygiene practices. Therefore leading to a more long-lasting sleep quality improvement among shift nurses [29]. Also, the effect of mindfulness programs on improving sleep quality may vary depending on shift type and chronotype [52]. Thus, personalised interventions should be tailored to address sleep problems for each participant based on their work schedule, chronotype and sleep habits [53].

Aromatherapy was seen as a convenient way to provide immediate mood stabilising and stress relieving effect, using single or blended essential oils, such as lavender and bergamot oil [54]. When inhaled, aromatic particles in the essential oil reach the limbic systems via olfactory nerves, providing stress relief, muscle relaxing effect and improving sleep quality [55]. Some studies also administered aroma essential oil through massage, in which aromatic molecules were absorbed through the skin and achieve its therapeutic effect [30, 54]. While previous reviews showed that aromatherapy could effectively improve shift nurses’ sleep quality [25], there was no significant improvement found among shift nurses’ receiving aromatherapy in this meta-analysis. These mixed findings may be resulted from the type of aroma used, exposure duration and route of application [22, 55]. Therefore, future research was needed to determine the most suitable aromatherapy regimes for shift nurses.

Summarising the current trend of non-pharmacological interventions adopted among shift nurses, aromatherapy was more commonly used previously [25], while mindfulness programs gained popularity after the COVID-19 pandemic. Implementation of aromatherapy was challenging during COVID-19 due to the risk of virus transmission from nebulisation or physical contact. In contrast, mindfulness programs could be easily implemented through online platform to overcome social distancing barrier. Also, behavioural changes made during mindfulness programs seemed to provide a longer effect on the improvement of sleep quality [53] than aromatherapy, which emphasised the immediate symptomatic relief for insomnia. Moreover, although aromatherapy often uses ingredients extracted from natural plants and flowers, these essential oil extracts may cause adverse effects, such as allergies, dermatitis and eye irritation [56]. Given these advantages, mindfulness program seemed to emerge as a safer and sustainable intervention for improving shift nurses’ sleep quality with fewer adverse effects.

In the overall pooled meta-analysis, high heterogeneity was observed due to the varying measurement tools used across the included studies, with PSQI and ISI used most commonly. Comparing the above two assessment tools, the ISI emphasises the severity of insomnia while the PSQI focused on overall sleep quality, which could be a key difference contributing to high heterogeneity in this meta-analysis. Also, previous review suggested that some items in PSQI may not be suitable to be applied among shift nurses due to their irregular work schedule, for example, night-time sleep duration, daytime waking time and average time needed to fall asleep at night [25]. Therefore, future studies were needed to design a new measurement tool tailored for shift workers’ to accurately reflect their irregular sleep pattern.

This review had several limitations. First, it only included papers reported in English so relevant articles written in other languages were missed. Second, high heterogeneity was found in the overall pooled meta-analysis due to the difference in measurement tools used across the included studies. The meta-analysis for aromatherapy and exercise training may not be powerful enough to conclude their effectiveness as only two or three studies were available for analysis. Third, subgroup analyses were attempted but could not be performed according to risk of bias and study location due to insufficient studies in key subgroups. Fourth, this meta-analysis could not assess the long-term effect of non-pharmacological interventions on improving sleep quality as most of the included studies did not include follow-up evaluation after intervention. Fifth, many studies were excluded as they tended to investigate sleep quality for all shift workers but not exclusively nurses. Future studies should design non-pharmacological intervention specifically for nurses working in rotating shifts to reduce bias from the variability of job nature.

In conclusion, this meta-analysis reflected those non-pharmacological interventions, specifically mindfulness programs, were effective in improving shift nurses’ sleep quality, but could be limited due to low compliance. Mindfulness programs had increased popularity after the COVID-19 pandemic due to online self-administration, overcoming the barrier of social distancing. Therefore, future interventions could adopt personalised online mindfulness programs to accommodate work schedules and increase compliance. Aromatherapy did not significantly improve shift nurses’ sleep quality in this meta-analysis, unlike a previous review [25], possibly due to a different aroma regime, which needs to be examined in future research. Moreover, to prevent high heterogeneity in future studies, a new assessment tool of sleep quality, tailor-made for shift workers, may be needed due to irregular sleep patterns.

## Supplementary Material

kqag085_Supplementary_Data
