# Supplementary material for: Non-pharmacological intervention for shift nurses’ sleep: systematic review and meta-analysis
Source: Occup Med (Lond). 2026 Sep 3;76(6):442–52. doi: 10.1093/occmed/kqag085 (PMC13673503; doi:10.1093/occmed/kqag085)
Supplement: Supplementary file 1 [file kqag085_supplementary_data.zip › Supplementary File S1.docx]

Supplementary File S1: Searching Strategies

1. **PubMed**

| **Searching Strategies** | (“Sleep”[Mesh]) OR (“Sleep Quality”[Mesh]) OR (“Sleep Wake Disorders”[Mesh]) OR (“Sleep Disorders, Circadian Rhythm”[Mesh]) OR (“Dyssomnias”[Mesh]) OR (“Sleep Hygiene”[Mesh]) OR (“Sleep Duration”[Mesh]) OR (“sleep*” OR “sleep* quality” OR “sleep* health” OR “dyssomnias” OR “sleep* disorder*” OR “sleep wake disorder*” OR “sleep-wake disorder*” OR “sleep* deprivation*” OR “sleep* deficien*” OR “insomnia*” OR “sleep* hygiene” OR “sleep* disturbance*” OR “sleep* impair*” OR “sleep* disruption*” OR “loss of sleep” OR “sleep* loss” OR “sleep* pattern*” OR “sleep* cycle” OR “sleep* duration*” OR “sleep* latency*” OR “circadian rhythm”)  **AND**  ("Nurses"[Mesh]) OR ("Nursing"[Mesh]) OR (“nurs*” OR “shift nurse*” OR “shift-nurse*”)  **AND**  ("Shift Work Schedule"[Mesh]) OR (“shift” OR “shift work*” OR “shiftwork” OR “shift-work” OR “night shift*” OR “night-shift” OR “nightwork” OR “rotat* shift*” OR “rotat* night shift*” OR “irregular work* hour*”) |
| --- | --- |
|  | **Second Round**  **AND**  Mindfulness  (“Mindfulness”[Mesh]) OR (“Meditation”[Mesh]) OR (“Relaxation Therapy”[Mesh]) OR (“mindfulness” OR “mindfulness-based” OR “cognitive behavioral therap*” OR “cbt” OR “cognitive behavioural therap*” OR “cognitive behavior therap*” OR “cognitive behaviour therap*” OR “meditation” OR “relaxation”)  Aromatherapy  (“Aromatherapy”[Mesh]) OR (“aroma” OR “aromatherapy*” OR “aroma therap*” OR “aroma-therap*” OR “essential oil*”)  Education Program  (“education” OR “education* program*” OR “workshop*” OR “training program*”)  Exercise  (“Exercise”[Mesh]) OR (“Exercise Therapy”[Mesh]) OR (“exercise” OR “exercise therap*” OR “exercise training” OR “physical activit*” OR “physical training”)  Chinese Medicine  (“Acupressure”[Mesh]) OR (“Chinese medic*” OR “acupressure “ OR “acupoint therapy” OR “acupoint pressure” OR “acupressure massage”)  Light therapy  (“Phototherapy”[Mesh]) OR (“light” OR “light therapy” OR “light treatment” or “phototherapy”) |
| **Filter** | - Publication Year: 2014-2024 |

2. **MEDLINE (via Ovid SP)**

| **Searching Strategies** | (“sleep*” OR “sleep* quality” OR “sleep* health” OR “dyssomnias” OR “sleep* disorder*” OR “sleep wake disorder*” OR “sleep-wake disorder*” OR “sleep* deprivation*” OR “sleep* deficien*” OR “insomnia*” OR “sleep* hygiene” OR “sleep* disturbance*” OR “sleep* impair*” OR “sleep* disruption*” OR “loss of sleep” OR “sleep* loss” OR “sleep* pattern*” OR “sleep* cycle” OR “sleep* duration*” OR “sleep* latency*” OR “circadian rhythm”)  **AND**  (“nurs*” OR “shift nurse*” OR “shift-nurse*”)  **AND**  (“shift” OR “shift work*” OR “shiftwork” OR “shift-work” OR “night shift*” OR “night-shift” OR “nightwork” OR “rotat* shift*” OR “rotat* night shift*” OR “irregular work* hour*”) |
| --- | --- |
|  | **Second Round**  **AND**  Mindfulness  (“mindfulness” OR “mindfulness-based” OR “cognitive behavioral therap*” OR “cbt” OR “cognitive behavioural therap*” OR “cognitive behavior therap*” OR “cognitive behaviour therap*” OR “meditation” OR “relaxation”)  Aromatherapy  (“aroma” OR “aromatherapy*” OR “aroma therap*” OR “aroma-therap*” OR “essential oil*”)  Education Program  (“education” OR “education* program*” OR “workshop*” OR “training program*”)  Exercise  (“exercise” OR “exercise therap*” OR “exercise training” OR “physical activit*” OR “physical training”)  Chinese Medicine  (“Chinese medic*” OR “acupressure “ OR “acupoint therapy” OR “acupoint pressure” OR “acupressure massage”)  Light therapy  (“light” OR “light therapy” OR “light treatment” or “phototherapy”) |
| **Filter** | - Publication Year: 2014-2024 |

3. **CINAHL Ultimate (via Ebscohost)**

| **Searching Strategies** | (MH "Sleep+") OR (MH "Sleep Hygiene") OR (MH "Sleep Deprivation") OR (MH "Sleep Quality") OR (MH "Sleep Duration") OR (MH "Sleep Disorders") OR (MH "Sleep Disorders, Circadian Rhythm+") OR (MH "Sleep Latency") OR (“sleep*” OR “sleep* quality” OR “sleep* health” OR “dyssomnias” OR “sleep* disorder*” OR “sleep wake disorder*” OR “sleep-wake disorder*” OR “sleep* deprivation*” OR “sleep* deficien*” OR “insomnia*” OR “sleep* hygiene” OR “sleep* disturbance*” OR “sleep* impair*” OR “sleep* disruption*” OR “loss of sleep” OR “sleep* loss” OR “sleep* pattern*” OR “sleep* cycle” OR “sleep* duration*” OR “sleep* latency*” OR “circadian rhythm”)  **AND**  (MH "Nurses+") OR (“nurs*” OR “shift nurse*” OR “shift-nurse*”)  **AND**  (MH "Shiftwork") OR (MH "Shift Workers") OR (“shift” OR “shift work*” OR “shiftwork” OR “shift-work” OR “night shift*” OR “night-shift” OR “nightwork” OR “rotat* shift*” OR “rotat* night shift*” OR “irregular work* hour*”) |
| --- | --- |
|  | **Second Round**  **AND**  Mindfulness  (MH "Cognitive Therapy+") OR (“mindfulness” OR “mindfulness-based” OR “cognitive behavioral therap*” OR “cbt” OR “cognitive behavioural therap*” OR “cognitive behavior therap*” OR “cognitive behaviour therap*” OR “meditation” OR “relaxation”)  Aromatherapy  (MH "Essential Oils+") OR (MH "Aromatherapy") OR (“aroma” OR “aromatherapy*” OR “aroma therap*” OR “aroma-therap*” OR “essential oil*”)  Education Program  (“education” OR “education* program*” OR “workshop*” OR “training program*”)  Exercise  (MH "Exercise+") OR (“exercise” OR “exercise therap*” OR “exercise training” OR “physical activit*” OR “physical training”)  Chinese Medicine  (MH "Acupressure+") OR (MH "Massage Therapy") OR (MH "Massage+") OR (MH "Acupuncture Points") OR (“Chinese medic*” OR “acupressure “ OR “acupoint therapy” OR “acupoint pressure” OR “acupressure massage”)  Light therapy  (MH "Phototherapy") OR (“Phototherapy”[Mesh]) OR (“light” OR “light therapy” OR “light treatment” or “phototherapy”) |
| **Filter** | - Publication Date: 2014-2024 - English Language |

4. **EMBASE (via Ovid SP)**

| **Searching Strategies** | (“sleep*” OR “sleep* quality” OR “sleep* health” OR “dyssomnias” OR “sleep* disorder*” OR “sleep wake disorder*” OR “sleep-wake disorder*” OR “sleep* deprivation*” OR “sleep* deficien*” OR “insomnia*” OR “sleep* hygiene” OR “sleep* disturbance*” OR “sleep* impair*” OR “sleep* disruption*” OR “loss of sleep” OR “sleep* loss” OR “sleep* pattern*” OR “sleep* cycle” OR “sleep* duration*” OR “sleep* latency*” OR “circadian rhythm”)  **AND**  (“nurs*” OR “shift nurse*” OR “shift-nurse*”)  **AND**  (“shift” OR “shift work*” OR “shiftwork” OR “shift-work” OR “night shift*” OR “night-shift” OR “nightwork” OR “rotat* shift*” OR “rotat* night shift*” OR “irregular work* hour*”) |
| --- | --- |
|  | **Second Round**  **AND**  Mindfulness  (“mindfulness” OR “mindfulness-based” OR “cognitive behavioral therap*” OR “cbt” OR “cognitive behavioural therap*” OR “cognitive behavior therap*” OR “cognitive behaviour therap*” OR “meditation” OR “relaxation”)  Aromatherapy  (“aroma” OR “aromatherapy*” OR “aroma therap*” OR “aroma-therap*” OR “essential oil*”)  Education Program  (“education” OR “education* program*” OR “workshop*” OR “training program*”)  Exercise  (“exercise” OR “exercise therap*” OR “exercise training” OR “physical activit*” OR “physical training”)  Chinese Medicine  (“Chinese medic*” OR “acupressure “ OR “acupoint therapy” OR “acupoint pressure” OR “acupressure massage”)  Light therapy  (“light” OR “light therapy” OR “light treatment” or “phototherapy”) |
| **Filter** | - Publication Year: 2014-2024 |

5. **PsycINFO (via Ovid SP)**

| **Searching Strategies** | (“sleep*” OR “sleep* quality” OR “sleep* health” OR “dyssomnias” OR “sleep* disorder*” OR “sleep wake disorder*” OR “sleep-wake disorder*” OR “sleep* deprivation*” OR “sleep* deficien*” OR “insomnia*” OR “sleep* hygiene” OR “sleep* disturbance*” OR “sleep* impair*” OR “sleep* disruption*” OR “loss of sleep” OR “sleep* loss” OR “sleep* pattern*” OR “sleep* cycle” OR “sleep* duration*” OR “sleep* latency*” OR “circadian rhythm”)  **AND**  (“nurs*” OR “shift nurse*” OR “shift-nurse*”)  **AND**  (“shift” OR “shift work*” OR “shiftwork” OR “shift-work” OR “night shift*” OR “night-shift” OR “nightwork” OR “rotat* shift*” OR “rotat* night shift*” OR “irregular work* hour*”) |
| --- | --- |
|  | **Second Round**  **AND**  Mindfulness  (“mindfulness” OR “mindfulness-based” OR “cognitive behavioral therap*” OR “cbt” OR “cognitive behavioural therap*” OR “cognitive behavior therap*” OR “cognitive behaviour therap*” OR “meditation” OR “relaxation”)  Aromatherapy  (“aroma” OR “aromatherapy*” OR “aroma therap*” OR “aroma-therap*” OR “essential oil*”)  Education Program  (“education” OR “education* program*” OR “workshop*” OR “training program*”)  Exercise  (“exercise” OR “exercise therap*” OR “exercise training” OR “physical activit*” OR “physical training”)  Chinese Medicine  (“Chinese medic*” OR “acupressure “ OR “acupoint therapy” OR “acupoint pressure” OR “acupressure massage”)  Light therapy  (“light” OR “light therapy” OR “light treatment” or “phototherapy”) |
| **Filter** | - Publication Year: 2014-2024 |

6. **Scopus**

| **Searching Strategies** | (“sleep*” OR “sleep* quality” OR “sleep* health” OR “dyssomnias” OR “sleep* disorder*” OR “sleep wake disorder*” OR “sleep-wake disorder*” OR “sleep* deprivation*” OR “sleep* deficien*” OR “insomnia*” OR “sleep* hygiene” OR “sleep* disturbance*” OR “sleep* impair*” OR “sleep* disruption*” OR “loss of sleep” OR “sleep* loss” OR “sleep* pattern*” OR “sleep* cycle” OR “sleep* duration*” OR “sleep* latency*” OR “circadian rhythm”)  **AND**  (“nurs*” OR “shift nurse*” OR “shift-nurse*”)  **AND**  (“shift” OR “shift work*” OR “shiftwork” OR “shift-work” OR “night shift*” OR “night-shift” OR “nightwork” OR “rotat* shift*” OR “rotat* night shift*” OR “irregular work* hour*”) |
| --- | --- |
|  | **Second Round**  **AND**  Mindfulness  (“mindfulness” OR “mindfulness-based” OR “cognitive behavioral therap*” OR “cbt” OR “cognitive behavioural therap*” OR “cognitive behavior therap*” OR “cognitive behaviour therap*” OR “meditation” OR “relaxation”)  Aromatherapy  (“aroma” OR “aromatherapy*” OR “aroma therap*” OR “aroma-therap*” OR “essential oil*”)  Education Program  (“education” OR “education* program*” OR “workshop*” OR “training program*”)  Exercise  (“exercise” OR “exercise therap*” OR “exercise training” OR “physical activit*” OR “physical training”)  Chinese Medicine  (“Chinese medic*” OR “acupressure “ OR “acupoint therapy” OR “acupoint pressure” OR “acupressure massage”)  Light therapy  (“light” OR “light therapy” OR “light treatment” or “phototherapy”) |
| **Filter** | - Publication Year: 2014-2024 - English |

7. **Cochrane Central Register of Controlled Trials (CENTRAL)**

| **Searching Strategies** | **#1:** MeSH descriptor: [Sleep] explode all trees  **#2:** MeSH descriptor: [Sleep Wake Disorders] explode all trees  **#3:** (sleep* OR sleep* quality OR sleep* health OR dyssomnias OR sleep* disorder* OR sleep wake disorder* OR sleep-wake disorder* OR sleep* deprivation* OR sleep* deficien* OR insomnia* OR sleep* hygiene OR sleep* disturbance* OR sleep* impair* OR sleep* disruption* OR loss of sleep OR sleep* loss OR sleep* pattern* OR sleep* cycle OR sleep* duration* OR sleep* latency* OR circadian rhythm OR fatigue OR stress*)  **#4:** MeSH descriptor: [Nurses] explode all trees  **#5:** MeSH descriptor: [Nursing] explode all trees  **#6:** (nurs* OR shift nurse* OR shift-nurse*)  **#7:** MeSH descriptor: [Shift Work Schedule] explode all trees  **#8:** (shift OR shift work* OR shift-work OR night shift* OR night-shift OR rotat* shift* OR rotat* night shift* OR shift-work* OR irregular work* hour*)  **#9:** #1 OR #2 OR #3  **#10:** #4 OR #5 OR #6  **#11:** #7 OR #8  **#12:** #9 AND #10 AND #11 |
| --- | --- |
|  | **Second Round**  **#1:** MeSH descriptor: [Sleep] explode all trees  **#2:** MeSH descriptor: [Sleep Wake Disorders] explode all trees  **#3:** (sleep* OR sleep* quality OR sleep* health OR dyssomnias OR sleep* disorder* OR sleep wake disorder* OR sleep-wake disorder* OR sleep* deprivation* OR sleep* deficien* OR insomnia* OR sleep* hygiene OR sleep* disturbance* OR sleep* impair* OR sleep* disruption* OR loss of sleep OR sleep* loss OR sleep* pattern* OR sleep* cycle OR sleep* duration* OR sleep* latency* OR circadian rhythm)  **#4:** MeSH descriptor: [Nurses] explode all trees  **#5:** MeSH descriptor: [Nursing] explode all trees  **#6:** (nurs* OR shift nurse* OR shift-nurse*)  **#7:** MeSH descriptor: [Shift Work Schedule] explode all trees  **#8:** (shift OR shift work* OR shift-work OR night shift* OR night-shift OR rotat* shift* OR rotat* night shift* OR shift-work* OR irregular work* hour*)  Mindfulness  **#9:** (mindfulness OR mindfulness-based OR cognitive behavioral therap* OR cbt OR cognitive behavioural therap* OR cognitive behavior therap* OR cognitive behaviour therap* OR meditation OR relaxation)  **#10:** MeSH descriptor: [Mindfulness] explode all trees  **#11:** MeSH descriptor: [Cognitive Behavioral Therapy] explode all trees  **#12:** #1 OR #2 OR #3  **#13:** #4 OR #5 OR #6  **#14:** #7 OR #8  **#15:** #9 OR #10 OR #11  **#16:** #12 AND #13 AND #14 AND #15  Aromatherapy  **#9:** (aroma OR aromatherapy* OR aroma therap* OR aroma-therap* OR essential oil*)  **#10:** MeSH descriptor: [Aromatherapy] explode all trees  **#11:** #1 OR #2 OR #3  **#12:** #4 OR #5 OR #6  **#13:** #7 OR #8  **#14:** #9 OR #10  **#15:** #11 AND #12 AND #13 AND #14  Education Program  **#9:** (“education” OR “education* program*” OR “workshop*” OR “training program*”)  **#10:** MeSH descriptor: [Education] explode all trees  **#11:** #1 OR #2 OR #3  **#12:** #4 OR #5 OR #6  **#13:** #7 OR #8  **#14:** #9 OR #10  **#15:** #11 AND #12 AND #13 AND #14  Exercise  **#9:** (exercise OR exercise therap* OR exercise training OR physical activit* OR physical training)  **#10:** MeSH descriptor: [Exercise] explode all trees  **#11:** #1 OR #2 OR #3  **#12:** #4 OR #5 OR #6  **#13:** #7 OR #8  **#14:** #9 OR #10  **#15:** #11 AND #12 AND #13 AND #14  Chinese Medicine  **#9:** (Chinese medic* OR acupressure OR acupoint therapy OR acupoint pressure OR acupressure massage)  **#10:** MeSH descriptor: [Acupressure] explode all trees  **#11:** #1 OR #2 OR #3  **#12:** #4 OR #5 OR #6  **#13:** #7 OR #8  **#14:** #9 OR #10  **#15:** #11 AND #12 AND #13 AND #14  Light therapy  **#9:** (light OR light therapy OR light treatment or phototherapy)  **#10:** MeSH descriptor: [Phototherapy] explode all trees  **#11:** #1 OR #2 OR #3  **#12:** #4 OR #5 OR #6  **#13:** #7 OR #8  **#14:** #9 OR #10  **#15:** #11 AND #12 AND #13 AND #14 |
| **Filter** | - Original Publication Year: 2014-2024 - Trial - English |
