# Supplementary material for: Non-pharmacological intervention for shift nurses’ sleep: systematic review and meta-analysis
Source: Occup Med (Lond). 2026 Sep 3;76(6):442–52. doi: 10.1093/occmed/kqag085 (PMC13673503; doi:10.1093/occmed/kqag085)
Supplement: Supplementary file 1 [file kqag085_supplementary_data.zip › Supplementary File S2.docx]

Supplementary File S2: Data Extraction Table

| **Study Characteristics** | | **Intervention Characteristics** | | **Outcomes** | | **Limitations** | **Recommendations** |
| --- | --- | --- | --- | --- | --- | --- | --- |
| **Author (Year)** | (1) **Study Design** (2) **Study Location** (3) **Study Population** (4) **Study Duration** | **Intervention Group (IG)** | **Control Group (CG)** | **Outcome Measures** | **Results** |  |  |
| **Mindfulness Program** | | | | | | | |
| Al-Hammouri et al. (2023) | (1) Study Design:  Randomized controlled trial  (2) Study Location  Northern Jordan  (3) Study Population  Shift nurses working in hospital  (n=123)  (Mean Age: 30.90)  **IG:** n=60  **CG:** n=63  (4) Study Duration  4 weeks | Mindfulness Program   - Total 8 sessions - 60 mins per session - **Content:** Perform mindful exercises (e.g. stretching, body scan)   After Program   - Video clips for home practices - Daily reminder messages | No intervention | **Data Collection Timepoint**   - T0: baseline - T1: 5 weeks   (1) Sleep quality   - PSQI   (2) Sleep duration   - Average of 7 days period | (1) Sleep quality   - IG was significantly better than CG   [T0: (IG) 10.90±3.59 vs (CG) 10.93±3.72]  [T1: (IG) 8.58±3.15 vs (CG) 15.10±3.90]  [MD: (T1) -6.53; F(1,121)=29.71; p<0.001]  (2) Sleep duration   - No significant difference   [T0: (IG) 6.83±1.50 vs (CG) 6.94±1.70]  [T1: (IG) 7.37±1.50 vs (CG) 6.71±1.60]  [MD: (T1) 0.66; F(1,121)=1.09; p=0.30] | (1) Single setting in one hospital  (2) Recall bias – Rely on self-reported measurement  (3) Lack of follow-up data | (1) Further studies on dose-response relationship between intervention duration and outcome in different hospitals  (2) Objective measurements (e.g. actigraphy)  (3) Further studies to understand the maintenance of intervention effect through follow-up evaluation |
| Dahlgren et al.  (2022) | (1) Study Design:  Randomized controlled trial  (2) Study Location  Sweden  (3) Study Population  Newly graduated shift nurses working in hospital  (n=145)  **IG:**  T1: n=70  T2: n=58  Mean Age: 27.5  **CG:**  T1: n=75  T2: n=72  Mean Age: 27.0  (4) Study Duration  6 weeks | Proactive Recovery Program   - Group-administered - Total 3 sessions - 1 session every 2 weeks - Cognitive behavioral therapy + motivational interviewing - Focus on relieving stress, improving sleep quality, and recovering from fatigue - Psycho-educative material with group discussion   Support after sessions   - Written materials for each session - Access to online resources (ArturNurse) for evaluating fatigue level and managing sleep | Waitlist control | **Data Collection Timepoint**   - T0: baseline - T1: 1 month after intervention - T2: 6 months after intervention   (1) Sleep quality   - ISI - KSQ | (1) Sleep quality  (ISI)   - No significant group by time interaction, but showed trend of improvement in IG   [T0: (IG) 10.65±5.07 vs (CG) 10.74±5.09]  [T1: (IG) 8.83±4.52 vs (CG) 10.15±4.50]  [T2: (IG) 8.83±4.50 vs (CG) 10.58±4.58]  [Group x Time: F=2.57; p=0.08]  (KSQ)   - No significant group by time interaction   [T0: (IG) 4.07±1.00 vs (CG) 4.03±1.04]  [T1: (IG) 4.42±0.84 vs (CG) 4.23±0.87]  [T2: (IG) 4.34±0.84 vs (CG) 4.20±0.85]  [Group x Time: F=0.75; p=0.47] | (1) Low compliance on attending all 3 sessions  (2) Biased by self-enrollment and recruited participants with higher motivation | (1) Need to increase attendance at group sessions  (2) Mandatory participation to reduce bias during enrollment  (3) Can perform qualitative interviews during follow-up |
| Ell et al. (2024) | (1) Study Design:  Randomized controlled trial  (2) Study Location  Germany  (3) Study Population  Shift nurses diagnosed with shift work disorder and insomnia, working in hospital  (n=46)  (Mean Age: 39.7)  **IG:**  n=23  Mean Age: 39.4  **CG:**  n=23  Mean Age: 40.1  (4) Study Duration  8 weeks | SleepCare Program  **(1) Digital Cognitive Behavioral Therapy for Insomnia (CBT-I)**   - Total 6 modules (Internet)   - Written materials, videos, interactive exercises   - Contents related to sleep, shift work, cognitive therapy for relaxation and improving sleep hygiene - Individualized feedback from trained clinical psychologists after each module   (2) Digital sleep diary  (3) Reminder text message for inactivity more than 10 days | Waitlist control | **Data Collection Timepoint**   - T0: baseline - T1: 8 weeks - T2: 6 months after intervention   (1) Sleep quality   - ISI - Sleep efficacy from sleep diary (%) - Sleep efficacy from actigraphy (%) | (1) Sleep quality  (ISI)   - IG have significant improvement than CG   [T0: (IG) 15.5±4.5 vs (CG) 15.3±3.9]  [T1: (IG) 10.1±6.4 vs (CG) 14.6±3.5]  [T2: (IG) 9.4±6.0]  [Group x Time: ß=-4.73; SE=1.12; p<0.001]  (Sleep efficacy from sleep diary)   - IG have significant improvement than CG   [T0: (IG) 80.5±7.7 vs (CG) 84.6±13.9]  [T1: (IG) 85.6±9.8 vs (CG) 83.3±9.0]  [T2: (IG) 84.9±9.6]  [Group x Time: ß=0.07; SE=0.03; p=0.017]  (Sleep efficacy from actigraphy)   - No significant difference   [T0: (IG) 81.6±13.6 vs (CG) 84.9±15.9]  [T1: (IG) 86.3±9.1 vs (CG) 82.2±18.6]  [T2: (IG) 85.8±12.3]  [Group x Time: ß=0.07; SE=0.04; p=0.069] | (1) Small sample size  (2) Very different shift schedule | NA |
| Hayajneh et al. (2024) | (1) Study Design:  Randomized controlled trial  (2) Study Location  Jordan  (3) Study Population  Shift nurses working in hospital  (n=100)  Mean Age: 29.76  **IG:**  n=50  **CG:**  n=50  (4) Study Duration  4 weeks | Mindfulness Program   - Total 4 session - One session per week - Content:   - Introduction to mindfulness   - Meditation exercise | Placebo Program   - Total 4 session - Watch video related to mindfulness only | **Data Collection Timepoint**   - T0: baseline - T1: 4 weeks   (1) Sleep quality   - PSQI | (1) Sleep quality   - Both groups improved, but with no statistical difference   [p=0.067] | (1) Small sample size’ | (1) Larger sample size  (2) Longer intervention duration for stronger effect’ |
| Nourian et al. (2021) | (1) Study Design:  Randomized controlled trial  (2) Study Location  Iran  (3) Study Population  Shift nurses working in hospital  (n=41)  Mean Age: 35.60  **IG:**  n=20  **CG:**  n=21  (4) Study Duration  7 weeks | Online Mindfulness-based Stress Reduction (MBSR) Program   - Total 7 session - One session per week - Conducted through WhatsApp - Material content:   - Basic knowledge of mindfulness   - Yoga and meditation - Logbook for reflection on mindfulness and meditation exercise - Text messaging for group and individual follow-up | Placebo Program   - Provide video related to COVID-19 care only | **Data Collection Timepoint**   - T0: baseline - T1: 7 weeks   (1) Sleep quality   - PSQI | (1) Sleep quality   - No significant difference between IG & CG   [IG: (T0) 9.90±2.48 vs (T1) 9.33±3.15; p=0.084]  [CG: (T0) 9.40±2.30 vs (T1) 10.6±2.49; p=0.001]  [p=0.105] | (1) Small sample size  (2) No long-term effect was measured | (1) Collect follow-up data to assess long-term effect on MBSR |
| Prasetyo et al. (2024) | (1) Study Design:  Quasi-experimental trial  (2) Study Location  Indonesia  (3) Study Population  Shift nurses working in hospital  (n=38)  (4) Study Duration  4 weeks | Spiritual Relaxation Therapy   - Total 6 session - 2 session per week (30 mins) - Breathing exercise + Meditation | Placebo Education Program on benefit of sleep | **Data Collection Timepoint**   - T0: baseline - T1: 1 week after program   (1) Sleep quality   - PSQI | (1) Sleep quality   - IG have significant improvement than CG   [T0: (IG) Mild (73.7%), Moderate (36.3%) vs (CG) Mild (68.4%), Moderate (31.6%)]  [T1: (IG) Mild (36.8%), Good (63.2%) vs (CG) Mild (78.9%), Moderate (21.2%)]  [p=0.001] | NA | NA |
| Zhou et al. (2022) | (1) Study Design:  Randomized controlled trial  (2) Study Location  China  (3) Study Population  Shift nurses with chronic insomnia working in hospital at airport site  (n=118)  **IG:**  n=60  Mean Age: 31.0  **CG:**  n=58  Mean Age: 29.6  (4) Study Duration  6 weeks | e-aid Cognitive Behavioral Therapy (eCBT-I)   - Total 6 modules - Content: management of insomnia (relaxation training, cognitive therapy, relieve anxiety) - Follow-up by professional therapists   - Weekly treatment plan by reviewing sleep reports | No intervention | **Data Collection Timepoint**   - T0: baseline - T1: 6 weeks   (1) Sleep quality   - PSQI - ISI | (1) Sleep quality  (PSQI)   - IG: Significant improvement   [IG: (T0) 10.4±3.5 vs (T1) 5.9±3.9; p<0.05]   - CG: No significant difference   [CG: (T0) 10.0±3.1 vs (T1) 9.1±3.9]   - IG have significant improvement than CG   [p<0.05]  (ISI)   - IG: Significant improvement   [IG: (T0) 12.4±4.7 vs (T1) 6.7±4.5; p<0.05]   - CG: No significant difference   [CG: (T0) 11.8±3.4 vs (T1) 10.6±4.1]   - IG have significant improvement than CG   [p<0.05] | (1) Mostly all women 🡪 not generalizable | (1) Should also long-term effect of CBT |
| **Aromatherapy** | | | | | | | |
| Chang et al. (2017) | (1) Study Design:  Randomized controlled trial  (2) Study Location  Taiwan  (3) Study Population  Female shift nurses with PSQI≥5 working in hospital  (n=50)  **IG:**  n=27  Mean Age: 28.36  **CG:**  n=23  Mean Age: 30.55  (4) Study Duration  4 weeks | Aromatherapy Massage   - Total 4 sessions (once a week) - 1 hour per session - 5 mins: laid down in aromatherapy room with soothing background music - 25 mins: perform aromatherapy massage with essential oil and massage oil made from sweet marjoram - 30 mins: rest and drink warm water | Placebo Control   - Total 4 sessions (once a week) - 1 hour per session - Laid down in a room with soothing background music only | **Data Collection Timepoint**   - T0: baseline - T1: 4 weeks   (1) Sleep quality   - PSQI - Subjective sleep quality (1-5; 1🡪very poor, 5🡪very good) | (1) Sleep quality  (PSQI)   - IG: Significant improvement   [IG: (T0) 8.89±2.45 vs (T1) 7.63±2.22; p=0.000]   - CG: No significant difference   [CG: (T0) 8.08±2.31 vs (T1) 7.58±1.92; p=0.191]   - No significant difference between IG & CG on the change of PSQI score   [Group x Time: Wald Chi-square=0.33; p=0.565]  (Subjective sleep quality)   - IG: Significant improvement reported in aromatherapy room, but no significant difference at home/dorm   [IG in aromatherapy room: (T0) 2.99±0.75 vs (T1) 3.57±0.63; p=0.009]  [IG at home/dorm: (T0) 3.35±0.70 vs (T1) 3.41±0.83; p=0.715]   - CG: No significant difference reported in aromatherapy room or at home/dorm   [CG in aromatherapy room: (T0) 2.97±0.72 vs (T1) 2.97±0.43; p=0.959]  [CG at home/dorm: (T0) 3.31±0.77 vs (T1) 3.39±0.90; p=0.795] | (1) Same environment with soothing music for both groups may affect the results  (2) Limited to one medical center | (1) Use control group with no intervention  (2) Larger sample size with more medical center involved |
| Ghods et al. (2016) | (1) Study Design:  Randomized controlled trial  (2) Study Location  Iran  (3) Study Population  Shift nurses working in hospital  (n=78)  **IG:**  n=39  Mean Age: 34.87  **CG:**  n=39  Mean Age: 36.49  (4) Study Duration  4 weeks | Aromatherapy   - Before shift: prepare 0.5 mL lavender oil into a container - During shift: carry aroma essential oil (around 20cm from their nose) for 2 hours | Placebo group   - Use sesame oil | **Data Collection Timepoint**   - T0: baseline - T1: 2 weeks - T2: 4 weeks   (1) Sleep quality   - PSQI | (1) Sleep quality   - No significant difference between 2 groups   [T0: (IG) 7.79±3.78 vs (CG) 7.44±4.24; p=0.694]  [T1: (IG) 5.97±2.51 vs (CG) 6.15±3.39; p=0.791]  [T2: (IG) 5.69±2.29 vs (CG) 6.05±2.81; p=0.539]   - Significant improvement along time in both groups   (IG) [T0 to T1: p<0.001; T0 to T2: p<0.001; T1 to T2: p=0.232]  (CG) [T0 to T1: p=0.005; T0 to T2: p=0.015; T1 to T2: p=0.599] | NA | NA |
| Karimi et al. (2024) | (1) Study Design:  Randomized controlled trial  (2) Study Location  Iran  (3) Study Population  Shift nurses working in intensive care unit  (n=84)  **IG:**  n=42  Mean Age: 31.6  **CG:**  n=42  Mean Age: 31.14  (4) Study Duration  3 work shifts | Aromatherapy   - Before shift: put one drop of P. graveolens essential oil into a dark linen pad and place it on the mask - During shift: carry aroma essential oil for 20mins twice in a shift | Placebo group   - Use sunflower oil | **Data Collection Timepoint**   - T0: baseline - T1: 2 weeks - T2: 4 weeks   (1) Sleep quality  Verran and Snyder-Halpern (VSH) Sleep Scale | (1) Sleep quality   - No significant difference between 2 groups and no significant within-group effect   [IG: (T0) 72.53±18.66 vs (T1) 77.58±13.53 vs (T2) 80.53±16.09; p=0.25]  [CG: (T0) 78.23±12.55 vs (T1) 79.58±11.8 vs (T2) 78.57±11.42; p=0.577]  [Group: p=0.458]  [Time x Group: 0.054] | (1) Difficult to perform sampling for 3 consecutive shifts  (2) Difficult to blinding due odor from aroma | NA |
| **Exercise Training** | | | | | | | |
| Baek et al. (2022) | (1) Study Design:  Quasi-experimental trial  (2) Study Location  South Korea  (3) Study Population  Shift nurses working in hospital with at least 3 months experience  (n=49)  **IG:**  n=25  Mean Age: 26.2  **CG:**  n=24  Mean Age: 28.8  (4) Study Duration  18 weeks | Smartphone-based Home Workout Program   - For 12 weeks - Used mobile apps with exercise and stretching modules - 1-2 workout daily for 3-5 days weekly   Text-message Counselling   - For 12 weeks - Once per week - Separated participants into 2 groups   - Group 1: contemplation, preparation stage   - Group 2: action, maintenance stage - Used counselling guideline according to groups   Poster   - For 18 weeks - Displayed in nurses’ office - Encouraged exercise by reminding benefits and coping strategies of exercise - Changed content every 2 weeks   Exercise Equipment   - Supplied every 4 weeks | No intervention | **Data Collection Timepoint**   - T0: baseline - T1: 6 weeks - T2: 12 weeks - T3: 18 weeks   (1) Sleep disturbance   - ISI | (1) Sleep disturbance   - IG was significantly lower than CG   [T0: (IG) 11.5±7.0 vs (CG) 11.8±6.8]  [T1: (IG) 7.9±5.8 vs (CG) 12.0±6.6]  [T2: (IG) 6.1±4.5 vs (CG) 11.5±7.1]  [T3: (IG) 7.1±5.0 vs (CG) 11.7±6.4]  [Group: F=5.32; p=0.03]  [Group x Time: F=5.41; p<0.01] | (1) Lack of generalizability: one hospital only  (2) No randomization 🡪 lower internal validity  (3) Response bias 🡪 self-reported | (1) Larger sample size with more organizations  (2) Random allocation  (3) Objective evaluation (e.g. use of device)  (4) Longer follow-up |
| Ha et al. (2022) | (1) Study Design:  Cluster randomized controlled trial  (2) Study Location  Korea  (3) Study Population  Shift nurses working in hospital  (n=57)  **IG:**  n=30  Mean Age: 27.63  **CG:**  n=27  Mean Age: 27.03  (4) Study Duration  12 weeks | Mobile Wellness Program  **(1) Fitbit**   - Self-monitoring physical activity and sleep pattern   **(2) Online group exercise**   - Through Zoom - 2 times a week (1 hour per session) - Live interaction with instructors and get personal feedback - Exercise video uploaded to Kakao Talk   **(3) Online health couching**   - Through Kakao Talk - Set goals, monitor progress, and discuss barriers faced   **(4) Motivational text message** | Received fit bit for self-monitoring only | **Data Collection Timepoint**   - T0: baseline - T1: 12 weeks   (1) Sleep quality   - PSQI | (1) Sleep quality   - No significant difference in total score   [IG: (T0) 9.23±3.18 vs (T1) 7.50±2.95; MD= -1.70±3.21]  [CG: (T0) 8.73±3.02 vs (T1) 8.53±2.82; MD= -0.22±4.04]  [t=-1.53, p=0.13]   - IG have significantly more improved than CG in some components of PSQI   [Subjective sleep quality: t=-2.29, p=0.025]  [Sleep disturbance: t=-2.00, p=0.049]  [Daytime dysfunction: t=-2.04, p=0.045] | (1) Small sample size  (2) Lack of generalizability: involving one hospital only  (3) All female nurses | NA |
| **Sleep Hygiene Education Program** | | | | | | | |
| Booker et al. (2022) | (1) Study Design:  Randomized controlled trial  (2) Study Location  Australia  (3) Study Population  Shift nurses working in hospital  (n=149)  (Mean Age: 34.66)  **IG:**  n=79  Mean Age: 33.42  **CG:**  n=70  Mean Age: 36.06  (4) Study Duration  8 weeks | One-to-one Couching Program   - Education on sleep hygiene   - Basic knowledge of sleep   - Napping and scheduled sleep   - Light exposure, caffeine consumption | Placebo Education Program on low GI diet | **Data Collection Timepoint**   - T0: baseline - T1: 10 weeks (2 weeks after intervention)   (1) Sleep Hygiene   - SHI   (2) Sleep disturbance   - ISI | (1) Sleep Hygiene   - No significant Program x Time Interaction   [p=0.981]  (2) Sleep disturbance   - Both groups have significant improvement   [Cohen’s d: (IG) -1.20 vs (CG) -1.05]   - No significant Program x Time Interaction   [p=0.477] | (1) Low compliance  (2) Possible positive effect of low GI education on sleep quality  (3) Cross-contamination of subjects between wards | (1) Need to incorporate cognitive behavioral therapy  (2) Need to improve compliance on home-based recommendations  (3) Use no intervention control + avoid disclosure of baseline questionnaire results  (4) Cluster randomization to avoid cross-contamination of subjects between wards |
| Yazdi et al. (2017) | (1) Study Design:  Randomized controlled trial  (2) Study Location  Iran  (3) Study Population  Shift nurses with clinical insomnia working in hospital  (n=100)  (Mean Age: 29.8)  **IG:**  n=50  Mean Age: 30.2  **CG:**  n=50  Mean Age: 29.4  (4) Study Duration  1 session | 2-hour Training   - Education on sleep hygiene   - Sleep hygiene practice   - Sleep-wake schedule   - Avoid caffeine | No intervention | **Data Collection Timepoint**   - T0: baseline - T1: 1 month after training   (1) Sleep quality   - Persian version of ISI - PSQI | (1) Sleep quality  (ISI)   - IG have significant improvement than CG   [T1: (IG) 9.35±1.20 vs (CG) 15.30±2.70; p<0.001]  (PSQI)   - IG have significant improvement than CG   [T1: (IG) 5.41±0.90 vs (CG) 6.97±1.20; p=0.003] | (1) Small sample size  (2) Only include nurse with clinical insomnia  (3) Subjective measurement | (1) Involve more hospitals  (2) Include actigraphy as measurement |
| **Light Therapy** | | | | | | | |
| Cyr et al. (2023) | (1) Study Design:  Randomized controlled trial  (2) Study Location  Canada  (3) Study Population  Shift nurses with insomnia working in hospital  (n=57)  **IG:**  n=28  Mean Age: 27.96  **CG:**  n=29  Mean Age: 34.34  (4) Study Duration  20 days | Evening Light Exposure   - 10 days per period - **The night before night shift:**   - Stay up one hour later   - Sleep with eye mask - **The day of night shift:**   - Morning: wear sunglasses   - Late afternoon: nap if needed   - Before night shift: 40 mins light exposure at home with a portable light box - **After final night shift:**   - Limit sleep time to be shorter for better transition to daytime routine | Placebo Intervention on chrononutrition | **Data Collection Timepoint**   - T0: baseline (first 10 days) - T1: Second 10 days - T2: Third 10 days   (1) Sleep quality   - Sleep quality scale (0-6; 0🡪worst, 5🡪best) | (1) Sleep quality   - No significant improvement in both groups   [Period x Group: p=0.166] | (1) Low compliance of eye mask  (2) Self-report bias | (1) Use objective measurement (e.g. wristband sensors) |

PSQI: Pittsburgh Sleep Quality Index

ISI: Insomnia Severity Index

SHI: Sleep Hygiene Index

KSQ: Karolinska Sleep Questionnaire

IG: Intervention Group, CG: Control Group

MD: Mean Difference
