# Supplementary material for: Non-pharmacological intervention for shift nurses’ sleep: systematic review and meta-analysis
Source: Occup Med (Lond). 2026 Sep 3;76(6):442–52. doi: 10.1093/occmed/kqag085 (PMC13673503; doi:10.1093/occmed/kqag085)
Supplement: Supplementary file 1 [file kqag085_supplementary_data.zip › Supplementary File S4.docx]

Supplementary File S4: Subgroup Analysis for Overall Pooled Effect

1. Shift nurses with vs without sleep problem

2. Study published before vs after 2020
